# Low-dose amikacin in the treatment of Multidrug-resistant Tuberculosis (MDR-TB)

**DOI:** 10.1186/s12879-021-05947-6

**Published:** 2021-03-10

**Authors:** Natasha F. Sabur, Mantaj S. Brar, Lisa Wu, Sarah K. Brode

**Affiliations:** 1 Department of Respirology, St. Michael’s Hospital and West Park Healthcare Centre, Rm 6-049, 30 Bond St, Toronto, ON M5B 1W8 Canada; 2 Department of Surgery, University of Toronto, Toronto, Canada; 3 West Park Healthcare Centre, Toronto, Canada; 4 Department of Respirology, Toronto Western Hospital, West Park Healthcare Centre, and University of Toronto, Toronto, Canada

**Keywords:** Aminoglycosides, Therapeutic drug monitoring, Hearing loss, Tuberculosis, Pharmacokinetics, Pharmacodynamics

## Abstract

**Background:**

The World Health Organization recommends intravenous amikacin for the treatment of MDR-TB at a dose of 15 mg/kg. However, higher doses are associated with significant toxicity.

**Methods:**

Patients with MDR-TB treated at our institution receive amikacin at 8–10 mg/kg, with dose adjustment based on therapeutic drug monitoring. We conducted a retrospective cohort study of patients with MDR-TB who received amikacin between 2010 and 2016.

**Results:**

Forty-nine patients were included in the study. The median starting dose of amikacin was 8.9 mg/kg (IQR 8, 10), and target therapeutic drug levels were achieved at a median of 12 days (IQR 5, 26). The median duration of amikacin treatment was 7.2 months (IQR 5.7, 8), and median time to sputum culture conversion was 1 month (IQR 1,2). Six patients (12.2%) experienced hearing loss based on formal audiometry testing (95% CI 4.6–24.8%); 22.2% had subjective hearing loss (95% CI 11.2–37.1%) and 31.9% subjective tinnitus (95% CI 19.1–47.1%). Ten patients (23%) had a significant rise in serum creatinine (95% CI 11.8–38.6%), but only 5 patients had a GFR < 60 at treatment completion. 84% of patients had a successful treatment outcome (95% CI 84–99%).

**Conclusions:**

Low dose amikacin is associated with relatively low rates of aminoglycoside-related adverse events. We hypothesize that low-dose amikacin can be used as a safe and effective treatment for MDR-TB in situations where an adequate regimen cannot be constructed with Group A and B drugs, and where careful monitoring for adverse events is feasible.

## Background

Despite decades of efforts at eliminating tuberculosis (TB), an estimated 10 million people were infected with TB and over 1.2 million people died of TB in 2018 [1]. Multidrug-resistant tuberculosis (MDR-TB), defined by resistance to both isoniazid and rifampin, has emerged as a global epidemic. Until recently, injectable aminoglycosides formed a core component of the treatment regimen used to treat MDR-TB, and both amikacin and streptomycin remain on the list of medicines recommended for use in MDR-TB regimens according to World Health Organization (WHO) guidelines [2].

Updated WHO guidelines for the treatment of drug-resistant TB have classified amikacin as a Group C drug, in part due to the significant toxicity associated with its use [2]. Previously, intravenous amikacin was classified as a Group B drug and more widely used, with a recommended dose of 15 mg/kg to a maximum dose of 1000 mg/day [3]. However, aminoglycosides at high doses are associated with significant toxicity, including ototoxicity, vestibular toxicity, and nephrotoxicity, and the optimal dose of amikacin remains controversial [4].

Although data regarding the pharmacokinetic (PK) and pharmacodynamic (PD) parameters in TB are relatively scarce, there is emerging evidence to support the use of lower-dose aminoglycoside therapy in the treatment of MDR-TB. Aminoglycosides are known to show concentration-dependent activity and thus the C_max_/MIC (maximum concentration to minimum inhibitory concentration) ratio is thought to be the most relevant PK/PD parameter to monitor their efficacy [5]. *Van Altena* and colleagues reported using low-dose amikacin and kanamycin with drug dosing based on therapeutic drug monitoring, using a target C_max_/MIC ratio of > 20. Their patients received a median dose of 6.5 mg/kg, achieved a mean weighted C_max_/MIC ratio of 31.2 with amikacin, and had lower reported rates of ototoxicity, while still achieving good treatment outcomes [6].

Prior to the wide-spread use of bedaquiline and availability of all-oral regimens for treatment of MDR-TB, amikacin was a standard component of the MDR-TB regimen. In our TB referral hospital in Toronto, Canada, patients received amikacin at a dose of 8–10 mg/kg, with dose adjustment based on therapeutic drug monitoring using 30-min post-dose levels, targeting a C_max_/MIC ratio of 25–35. These targets were selected based upon recommended peak serum levels for nontuberculous mycobacterial infections [7] (using extended interval dosing) and gram negative infections [8] (using traditional dosing), given the lack of TB PK/PD data at the time of the study. Here we report our experience with patient tolerability, side-effects and treatment outcomes using this method of dosing and therapeutic drug monitoring.

## Methods

We conducted a retrospective cohort study of all patients with MDR-TB who received amikacin from 2010 to 2016, inclusive. The study was approved by our institutional research board. We included patients who met the following criteria: age > 18 years; confirmed MDR-TB on genotypic or phenotypic drug susceptibility testing; and received amikacin. Patients who had confirmed amikacin-resistance on drug susceptibility testing were excluded from the study.

### Data collection

Patient demographics at the time of treatment initiation, clinical details, microbiologic data, baseline serum creatinine, audiometry test results and subsequent treatment details were retrospectively abstracted from patient charts. Parameters such as dose per kg body weight and total cumulative dose were calculated using abstracted data. Clinical outcomes over a minimum 2-year follow-up period were recorded.

### Specimen culture and drug susceptibility testing

Cultures were performed, processed, and analyzed using the Bactec MGIT 960 system (Becton Dickinson Microbiology Systems). The first culture positive specimen from each patient routinely underwent phenotypic drug susceptibility testing to first line drugs. Specimens demonstrating multi-drug resistance also underwent phenotypic drug susceptibility testing to amikacin and other standard second-line medications at the Public Health Ontario Laboratory. Individual MICs for amikacin were not reported, however the laboratory reported susceptibility or resistance to amikacin using a critical concentration of 1.0 mg/L. Repeat drug susceptibility testing was routinely performed if specimens were still culture positive after 3 months of treatment, or at the request of the treating physician.

### Amikacin administration, therapeutic drug level monitoring, and pharmacokinetics (PK)

Amikacin was administered intravenously via peripheral inserted central catheter (PICC) line daily (7 days/week) for a minimum of 3 months. Thereafter, once sputum culture conversion had been achieved, administration was thrice weekly. Each amikacin dose was delivered over a 30-min period. The distribution phase of amikacin was predicted to be complete at 0.5 h following the end of infusion, as amikacin would be in first order elimination after this time point in accordance with aminoglycoside PK. Therefore, peak concentration levels were taken 30-min post drug infusion and steady state expected after the initial dose, as the half-life of amikacin is expected to be 2.5 h or less [9, 10]. Initial trough and peak concentration drug levels were drawn within 3 days of the start of amikacin administration [11]. Since the MIC for amikacin-sensitive isolates will be lower than the critical concentration and therefore less than 1, we conservatively set the MIC at 1, and doses were adjusted to target peak concentration of 25–30 mg/L with daily dosing and 30–35 mg/L with thrice weekly dosing, and thus C_max_/MIC ratios of 25–30 and 30–35, respectively. Follow-up therapeutic drug levels were drawn monthly, and doses adjusted accordingly.

### Adverse events and clinical outcomes

We monitored for ototoxicity using formal audiometry testing. Baseline audiometry was performed following amikacin initiation, and monthly audiometry testing was recommended thereafter for the duration of amikacin therapy. Testing was performed at 250, 500, 1000, 2000, 4000, 8000, 10,000 and 12,000 Hz. Hearing loss was defined as ≥20 dB decline in hearing threshold from baseline in either ear at any frequency [12]; severe hearing loss was defined as ≥70 dB decline in hearing threshold from baseline in either ear [13]. Renal toxicity was monitored with monthly serum creatinine testing. Nephrotoxicity was defined by ≥50% rise in serum creatinine from baseline at any point during amikacin treatment. Renal recovery was defined as serum creatinine less than 1.25x the baseline creatinine at the time of treatment completion. Patients were routinely asked about subjective hearing loss, tinnitus, and dizziness at clinic appointments.

End of treatment outcomes were defined according to the WHO [14], and included success (cure/treatment completion), treatment failure, death during treatment, and loss to follow up/not evaluated. The definition for treatment failure was modified slightly from the WHO such that a regimen change due to adverse events alone was not considered a failure. All patients who had outcomes evaluated without loss to follow up were followed for a minimum of 2-years post-treatment completion to assess for relapse.

### Statistical analysis

Descriptive statistics were calculated for baseline variables and treatment characteristics, reporting proportions and medians with interquartile range (IQR), as appropriate. For binary treatment outcomes, exact binomial confidence intervals were reported. We performed a priori exploratory analyses to assess whether age, diabetes, or cumulative dose of amikacin were associated with hearing loss using Fisher’s exact tests, and logistic models for categorical and continuous variables, respectively. We assessed whether nephrotoxic drugs were associated with nephrotoxicity using Fisher’s exact test; lastly, we assessed whether diabetes, low body mass index (< 18.5), nephrotoxicity, or hearing loss were associated with treatment success using Fisher’s exact test. Given the low event rate, no multivariable analysis was possible. All analyses were performed using Stata V15 (StataCorp, College Station, TX).

## Results

### Patient characteristics

Patient characteristics are described in Table 1. Forty-nine patients met inclusion criteria. 22 (45%) were female and the median age was 31 (IQR 27, 52). 41 (84%) patients had pulmonary disease. 10% of patients had pre-XDR and 1% had XDR-TB. 1 (2%) patient was co-infected with HIV and 9 (18%) had coexisting diabetes mellitus.

**Table 1:** Baseline characteristics (*N* = 49)

	Number (%) or Median (IQR) Patients Receiving Amikacin
Female	22 (45%)
Age	31 (27, 52)
BMI	20.7 (18.8, 22.8)
Country of Origin	
Africa	4 (8%)
Asia	40 (82%)
Eastern Europe	4 (8%)
Canada	1 (2%)
TB location	
Pulmonary	38 (78%)
Extra-pulmonary	8 (16%)
Both	3 (6%)
Drug-Susceptibility	
MDR-TB	43 (88%)
Pre-XDR	5 (10%)
XDR	1 (2%)
Co-morbidity	
HIV	1 (2%)
Diabetes mellitus	9 (18%)

*BMI* Body Mass Index, *TB* Tuberculosis, *MDR* Multidrug-resistant, *XDR* Extensively drug-resistant, *HIV* Human immunodeficiency virus

### MDR-TB treatment details

Treatment details are outlined in Tables 2 and 3. Per guideline recommendations at that time, our patients received 5 drugs in the intensive phase and 4 drugs in the continuation phase; however, due to drug intolerances, a median of 7 drugs (IQR 6, 8) were trialed in the intensive phase, and 5 drugs in the continuation phase (IQR 4, 5) of therapy. The median starting dose of daily amikacin was 8.9 mg/kg (IQR 8, 10) or 500 mg (IQR 450, 587.5). Target therapeutic drug levels with daily therapy (peak of 25–30 mg/L) were achieved at a median of 12 days (IQR 5, 26). The highest peak level recorded during treatment was a median of 34.8 (IQR 31.9, 37.6) during daily treatment. Patients were switched to thrice weekly dosing after a median of 139 days (IQR 92, 173). Median amikacin dose was 8.1 mg/kg (IQR 7.3, 9.2) or 487.5 mg (IQR 412.5, 550) during thrice weekly treatment, and amikacin was continued for a median of 115 days (IQR 94, 150) at this dose. 85.3% (SD 0.14) of levels recorded over the course of amikacin treatment were within therapeutic range. The median total treatment duration with amikacin was 7.2 months (IQR 5.7, 8); total MDR-TB treatment duration was a median of 22 months (IQR 20, 25).

**Table 2:** Drugs used in treatment regimen (*n* = 49)

Drug	Patients, n (%)
Amikacin	49 (100)
Ethambutol	29 (59.2)
Pyrazinamide	29 (59.2)
Rifabutin	3 (6.1)
Levofloxacin	7 (14.3)
Moxifloxacin	44 (89.8)
Clofazimine	44 (89.8)
Linezolid	26 (53.1)
Ethionamide	30 (61.2)
Cycloserine	30 (61.2)
Para-aminosalicylic acid	32 (65.3)
Imipenem	12 (24.5)
Amoxicillin-clavulanate	6 (12.2)
Bedaquiline	5 (10.2)
Delamanid	1 (2)

**Table 3:** Treatment details

	Median (IQR) or Mean +/− SD
Median number of drugs in intensive phase^a^	7 (6, 8)
Median number of drugs in continuation phase^a^	5 (4, 5)
Median duration of amikacin treatment	
Daily dosing (days)	139 (92, 173)
TIW dosing (days)	115 (94, 150)
Total duration (months)	7.2 (5.7, 8)
Median starting dose of amikacin (mg/kg)	
Daily	8.9 (8, 10)
TIW	8.1 (7.3, 9.2)
Median cumulative dose of amikacin (g)	80.6 (61.7, 100.4)
Proportion of drug levels within target range	89% (78–93%)
Median number of audiometry tests during amikacin treatment	3 (1, 5)
Median total treatment duration (months)	22 (20, 25)

*TIW* Three times weekly

^a^Patients routinely received 5 drugs in the intensive phase and 4 drugs in the continuation phase. However, many patients required medication adjustments due to drug intolerances. The number displayed represents the median number of drugs trialed in order to establish the patient on an appropriate regimen

### Adverse events

Details of adverse events are described in Table 4. The median number of audiometry tests performed during amikacin treatment was 3 (2, 5). 6 (12.2%) patients experienced hearing loss based on formal audiometry testing (95% CI 4.6–24.8%); 10 (22.2%) patients had subjective hearing loss (95% CI 11.2–37.1%). In those with audiometry-confirmed hearing loss, 5 patients (83.3%) experienced hearing loss in the high frequency range (> 2000 Hz) – 4 patients experienced hearing loss at 8000 Hz and 1 patient experienced hearing loss at 12000 Hz. 5 (10.2%) patients experienced hearing loss at the conventionally tested frequencies of 8000 Hz or less. Hearing loss was bilateral in 4 patients (66.7%). No patient in our cohort developed severe hearing loss. There was no association between hearing loss and presence of diabetes, age, age > 65, cumulative dose of amikacin or high cumulative amikacin dose (> 100 g, which corresponds to the 75th percentile for cumulative dose). 31.9% of patients experienced subjective tinnitus during treatment with amikacin (95% CI 19.1–47.1%), and 8.5% experienced subjective dizziness (95% CI 2.4–20.4%). A total of 10 patients (21%) discontinued amikacin prematurely due to some form of toxicity.

**Table 4:** Adverse events

	N (%) or Median (IQR)
Sensorineural Hearing Loss	6 (12.2%)
Bilateral	4/6 (66.7%)
High frequency (> 2000 Hz)	5/6 (83.3%)
Very high frequency (> 10,000 Hz)	1/6 (16.7%)
Severe hearing loss	0/6 (0%)
Subjective hearing loss	10 (22%)
Subjective tinnitus	15 (31.9%)
Subjective dizziness	4 (8.5%)
Nephrotoxicity	
Baseline serum creatinine	64 (51, 73)
Peak serum creatinine	90 (76, 106)
End of treatment serum creatinine	76 (64, 92)
Number of patients with nephrotoxicity	10/43 (23.3%)
% recovery by end of treatment	2/10 (20%)
GFR < 60 at end of treatment	5 (11.9%)
Proportion of patients who discontinued amikacin prematurely due to AE	21% (17–43%)

*GFR* Glomerular filtration rate, *AE* Adverse events

The median baseline serum creatinine was 64 (IQR 51, 73), and 10 (23%) patients had nephrotoxicity (95% CI 11.8–38.6%) to a median peak creatinine of 90 (76, 106). 20% of these patients had recovery of serum creatinine by the end of treatment. Much of the described nephrotoxicity was subclinical; only 5 patients had a GFR < 60 ml/min/m^2^ at the time of treatment completion. Patients receiving other nephrotoxic medications (ACE inhibitors, loop diuretics, antiretroviral therapy, or chemotherapy agents) were more likely to experience a significant rise in creatinine during amikacin treatment, and less likely to have recovery of renal function by the end of MDR-TB treatment (86% of patients not receiving other nephrotoxic medications had recovery of renal function vs. 40% of patients receiving nephrotoxic medications in addition to amikacin; *p* = 0.043). However, this observation was based on a small number of patients.

### Treatment outcomes

Treatment outcomes are detailed in Table 5. The median time to sputum culture conversion in those patients with pulmonary disease was 1 month (IQR 1,2). 84% of our patients had a successful treatment outcome (95% CI 84–99%). Six patients (12%) were lost to follow up or not evaluated, and 2 patients (4%) had poor treatment outcomes (1 treatment failure, 1 death). No patients experienced relapse. There was no relationship between pre-existing diabetes, HIV infection, or low BMI (< 18.5) and treatment outcomes; likewise, the development of renal failure or hearing loss during treatment was not correlated with treatment outcomes.

**Table 5:** Treatment outcomes

	Mean +/− SD or N (%)
Time to culture conversion (months)	1.7 ± 1.2
Successful treatment outcome (cure/treatment completion)	41 (84%)
Loss to follow up/not evaluated	6 (12%)
Treatment failure/relapse	1 (2%)
Death	1 (2%)

## Discussion

Prior to 2018, amikacin was considered a Group B drug and therefore widely used in MDR-TB treatment. The introduction of bedaquiline and all-oral regimens for MDR-TB treatment has relegated amikacin to a Group C drug. However, aminoglycosides remain important drugs in MDR-TB treatment, as they exhibit concentration-dependent bactericidal activity against *Mycobacterium tuberculosis* [15, 16] and are highly effective in the treatment of drug-resistant TB isolates. In a large individual patient data meta-analysis, the use of injectable amikacin in patients with susceptible strains was associated with greater treatment success [17]. Of all the group C medications classified in the newest WHO guidelines, amikacin has the lowest adjusted odds ratio for treatment failure/relapse versus treatment success; its placement lower down on the list of preferred medications is primarily related to other issues with its use, including difficulty of administration, adverse events, and need for clinical monitoring [2]. In our patient cohort, patients receiving low-dose amikacin with regular clinical monitoring had a lower rate of adverse events compared to that reported in the literature with standard dose therapy. We believe that in situations where an adequate regimen cannot be constructed with Group A and B drugs, low-dose amikacin remains a viable option for MDR-TB treatment.

Extended interval aminoglycoside dosing regimens are used to optimize bactericidal activity by achieving desired peak concentration (C_max_) to minimum inhibitory concentration (MIC) ratio [16]. C_max_/MIC ratio is determined to be the most relevant PK/PD parameter to monitor amikacin efficacy based on the principle of aminoglycoside concentration-dependent killing [4, 5]. Experimental data demonstrate that a C_max_/MIC ratio of at least 10 *at the site of infection* is necessary to optimize bactericidal activity for amikacin, with MIC ranging from 0.5 to 2 mg/L. [4, 18, 19] At our center, individual MICs for amikacin are not reported. However, the critical concentration for amikacin on the Bactec MGIT 960 system, used by the Public Health Ontario Laboratory, is known to be 1.0 [20]. Since the MIC for amikacin-sensitive isolates will be lower than the critical concentration and therefore less than 1, we conservatively set the MIC at 1 and used a serum drug level drawn 30-min after amikacin dose delivery to estimate the C_max_/MIC ratio. By this method, we targeted and achieved a serum C_max_/MIC of well over 10 (25–30 with daily dosing and 30–35 with thrice weekly dosing), while still delivering lower than conventional doses of amikacin and avoiding unnecessary toxicity. Van Altena et al targeted a serum C_max_/MIC of > 20 with amikacin, and achieved a mean weighted C_max_/MIC of 31.2 [6], very similar to those achieved by our method and with a similarly low rate of ototoxicity and good clinical outcomes. However, the penetration of aminoglycosides into lung tissue and other target organs is poorly understood, with variable estimates reported [4]. Therefore, the optimal serum C_max_/MIC ratio, or the ratio needed to achieve a C_max_/MIC of > 10 at the site of infection (usually the lung), remains unknown. Srivastava et al hypothesized that a C_max_/MIC ratio of 67–89 would be necessary to achieve the target C_max_/MIC ratio of 10.3 in the lung based on a hollow-fiber model, but this was based upon a reported serum to bronchial secretion ratio of 0.135 [19]. However, Sartre et al found a higher serum to bronchial secretion ratio of 0.3, which would necessitate a serum C_max_/MIC ratio of approximately 34, much closer to the targets we utilized [21]. More research into amikacin serum to target organ penetration is needed [4]. Our results, and those of Van Altena et al [6], suggest that the targets we utilized may be adequate in a high-resource setting when amikacin is administered as part of a strong, multi-drug regimen.

Ototoxicity remains one of the most debilitating side effects related to aminoglycoside use. In a similar setting to ours but using standard dose aminoglycosides, ototoxicity based on regular audiogram testing occurred in 18% of patients in the Netherlands [22] 28% in the United Kingdom [23] and 37% of patients in the United States [24] although the US cohort included patients both with TB and nontuberculous mycobacterial disease. Rates of ototoxicty as high as 62% have been reported in MDR-TB patients treated in limited-resource settings [25] and is known to occur more frequently in populations with high rates of HIV co-infection [26]. Hearing loss tends to occur first at high frequencies, higher than those for human speech, and thus requires frequent monitoring with audiograms for early detection. Additionally, it can progress even after aminoglycosides have been stopped, making early detection imperative to avoid significant morbidity [27]. Risk factors for hearing loss with aminoglycoside use include higher cumulative dose of treatment [28], HIV co-infection [29], and older age [30]. In our cohort, 12% of patients experienced hearing loss confirmed on audiometry testing and the majority of these were detected at high frequencies. Our audiometry lab tested patients up to 12,000 Hz, detecting subtle changes at high frequencies that would have otherwise been missed, and only 10% had hearing loss at the conventionally tested frequencies of 8000 Hz or less. None of our patients experienced severe hearing loss. However, we found subjective tinnitus to be a common complaint. The low cumulative dose of aminoglycoside therapy combined with the low rate of HIV co-infection in our cohort likely explains the relatively limited ototoxicity we observed compared to rates reported in the literature, however even with low-dose amikacin, otoxicity remains a concern.

Hair cell injury in the inner ear by aminoglycosides also results in vestibular toxicity, which can manifest as disequilibrium and dizziness [31, 32], and can occur in up to 20% of patients receiving these drugs intravenously [32]. Dizziness was an uncommon complaint experienced by less that 10% of our patients, although our assessments were based on patient-reported symptoms and may therefore have been underestimated.

Aminoglycosides are renally excreted and known to cause nephrotoxicity. This is triggered by uptake of drug by renal tubular cells after glomerular filtration, leading to intra-cellular accumulation and subsequent tubular necrosis [33]. Nephrotoxicity occurs in up to 10% of MDR-TB patients [22, 34], though typically is reversible once the drug is discontinued. Our cohort had a slightly higher rate of nephrotoxicity than that reported in the literature, though this may be related to the definition used, as many patients met our criteria for nephrotoxicity but had serum creatinine and GFR within the normal range at the time of treatment completion. Based on the small number of patients in our cohort, there seemed to be a higher risk of irreversible nephrotoxicity when amikacin was used in combination with other known nephrotoxic medications.

Our study has several limitations that require discussion. As this was a consecutive cohort study with no control group, we could not evaluate the treatment effectiveness of low-dose amikacin in comparison to regimens with standard dosing or regimens excluding amikacin entirely. In addition, our sample size was modest to estimate the proportion of patients with sensorineural hearing loss. Due to the fact that our Public Health laboratory does not report individual MICs for amikacin, we used critical concentration to estimate an MIC of 1 for amikacin susceptible isolates, and therefore likely underestimated our achieved C_max_/MIC ratios. Although monthly audiometry was routinely arranged for patients, a median of only 3 audiometry tests were administered despite a median treatment duration of 7 months of amikacin. Although we may have missed some patients with mild hearing loss, we believe that many patients did not attend audiology appointments because they did not have overt concerns with their hearing.

The treatment outcomes in our patient cohort were similar to those in other high-income settings [17] and in settings where standard dose aminoglycosides were routinely utilized [35, 36] and we found a lower rate of aminoglycoside-related adverse events than reported in the literature [22, 23, 25], suggesting that low-dose amikacin, when used with careful clinical monitoring, is relatively safe, yet still effective in the treatment of MDR-TB. When an adequate regimen cannot be constructed with Group A and B drugs, we believe that low-dose amikacin should be considered as a component of a multidrug treatment regimen, as long as adequate resources for monitoring are in place. While there remain significant limitations to the use of low-dose amikacin, including cost, difficulty of administration, and higher than desirable toxicity, our study suggests that a lower dose approach with therapeutic drug monitoring may limit toxicity, and that early detection of high frequency hearing loss enables discontinuation of amikacin, avoiding serious or debilitating hearing loss.

## Conclusions

Although recent guidelines have relegated amikacin to a group C drug, we believe that it remains an important treatment option for MDR-TB in patients with complex resistance patterns or those with toxicity to Group A and B drugs. We demonstrate that low-dose amikacin with dose adjustment based on therapeutic drug monitoring has fairly limited toxicity with good treatment outcomes. Future prospective trials evaluating lower dose aminoglycosides and therapeutic drug level monitoring-guided dosing in MDR-TB would be beneficial.

## Data Availability

The dataset used and analyzed during the current study are available from the corresponding author upon reasonable request.

## Abbreviations

TB: Tuberculosis
MDR-TB: Multidrug-resistant tuberculosis
WHO: World Health Organization
PK: Pharmacokinetics
PD: Pharmacodynamics
C_max_/MIC ratio: Maximum concentration to minimum inhibitory concentration ratio
PICC: Peripheral inserted central catheter
BMI: Body mass index
HIV: Human immunodeficiency virus
